# Polyamines in morphogenesis and development: a promising research area in seaweeds

**DOI:** 10.3389/fpls.2015.00027

**Published:** 2015-02-04

**Authors:** Manoj Kumar, C. R. K. Reddy, Peter J. Ralph

**Affiliations:** ^1^Institute of Plant Sciences, Agricultural Research Organization (ARO), The Volcani CenterBet Dagan, Israel; ^2^Discipline of Marine Biotechnology and Ecology, Central Salt and Marine Chemicals Research Institute, Council of Scientific and Industrial Research (CSIR)Bhavnagar, India; ^3^Plant Functional Biology and Climate Change Cluster (C3), University of Technology SydneySydney, NSW, Australia

**Keywords:** polyamines, spermine, seaweed, morphogenesis, reproduction and development, sporulation and carposporogenesis

## Introduction

**Figure 1:**
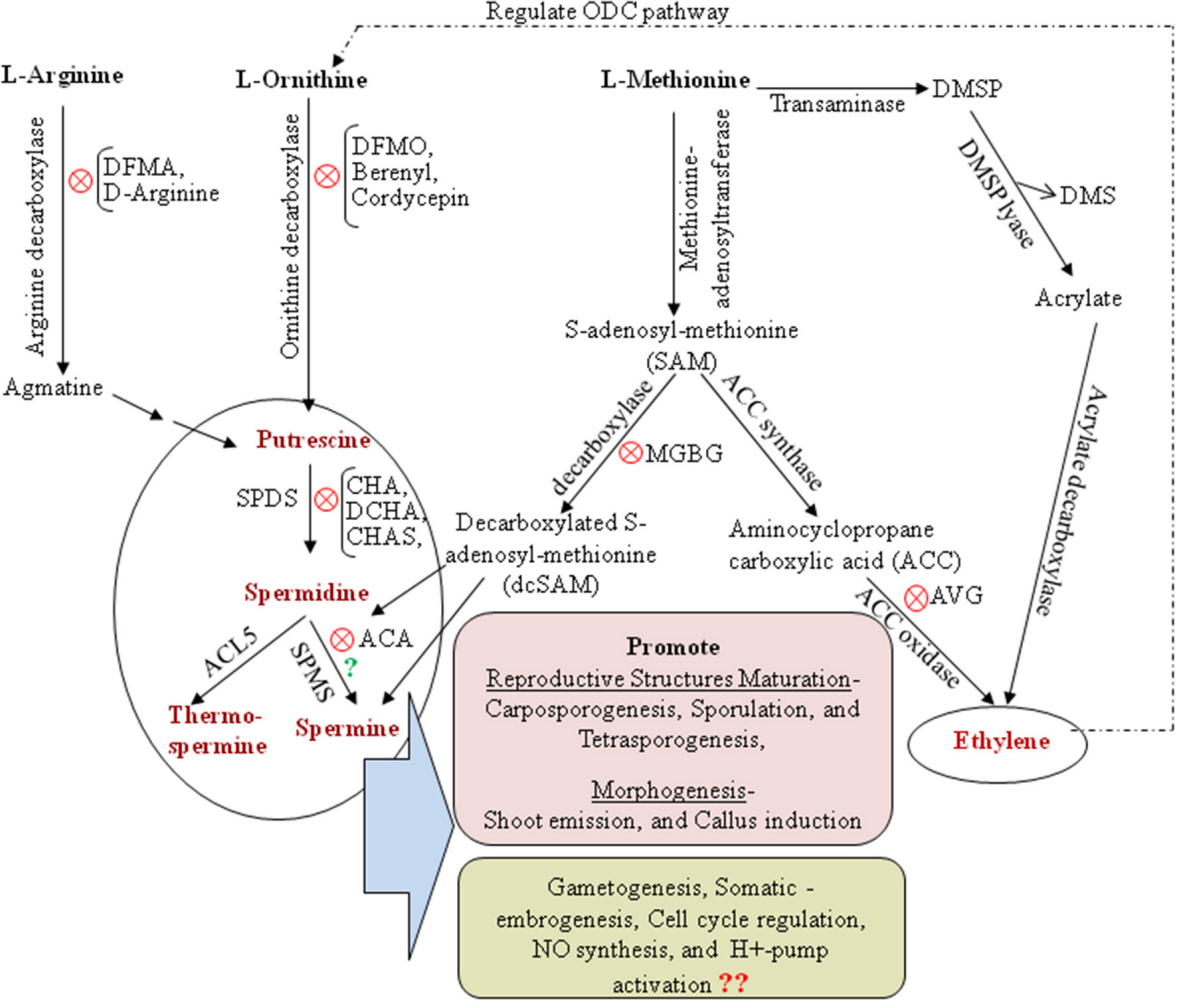
**Polyamine and ethylene biosynthetic pathway with special reference to seaweeds**. ODC, ornithine decarboxylase; DFMA, difluoromethylarginine; DFMO, difluoromethylornithine; DMSP, Dimethylsufoniopropionate; CHA, cyclohexylamine; CHAS, cyclohexylammonium sulfate; DCHA, dicyclohexylamine; ACA, 1′-acetoxychavicol acetate; MGBG, methylglyoxal bis-guanylhydrazone; AVG, aminoethoxyvinylglycine; NO, nitric oxide; SPDS, spermidine synthase; SPMS, spermine synthase; ACL5, acaulis5. Symbol “X” in red circle in several steps indicate the inhibitor for respective PA biosynthesis pathway. Symbol “?” in green questioned the existence of Spm biosynthesis pathway in seaweeds.

Polyamines (PAs), low molecular weight aliphatic amines, are ubiquitous in all living organisms except the archaeal methanogens and halophiles. In marine macroalgae, commonly known as seaweeds, the diamine putrescine (Put), triamine spermidine (Spd), and tetramine spermine (Spm) are the major PAs, although some brown and red seaweeds reported to have nor-spermine and nor-spermidine PAs. In plants, PAs have been shown to modulate a diverse range of biological processes including cell growth, development, and responses to various biotic and abiotic stresses (Minocha et al., 2014; Tiburcio et al., 2014 and references therein). Seaweeds share with land plants similar PA metabolic pathways (Figure 1). However, our understanding of the functionality of PAs in seaweed when compared to land plants has merely scratched the surface, though the existence of PAs in seaweeds was reported for over two decades ago (Baldini et al., 1994). So far, PA research in seaweeds has addressed their involvement in maturation of reproductive structures, morphogenesis (García-Jiménez et al., 1998; Marián et al., 2000; Guzmán-Urióstegui et al., 2002, 2012) and to some extent their response to abiotic stresses (Kumar et al., 2011, 2012, 2014). The progress on PA research in seaweed has been impeded mainly due to a lack of genomic information for seaweed species. In this brief article, we attempt to summarize PAs research into seaweed developmental biology and provide an in-depth analysis of findings to decipher PAs involvement in seaweeds development and morphogenesis with particular recognition of recently completed genome sequences of brown and red seaweeds. We also emphasize to re-investigate the existence and functionality of Spm in seaweeds as an inducer for maturing reproductive structures in context to the recent developments on tSpm (thermospermine) present throughout the whole plant kindgdom while Spm only in angiosperms.

## Occurrence of PAs and their role in morphogenesis, reproduction and development in seaweeds

Baldini et al. (1994) for the first time detected endogenous PAs, their uptake (passive and restricted to cell wall components) and transport (symplastic and stimulated by Ca^2+^) in the green seaweed *Ulva rigida*. However, it was Prof. Rafael Robaina's group who first identified PA's induced changes in growth (higher cell division and elongation rate) and morphogenesis (transforming carposporelings into morphogenic cell masses) in the red seaweed *Grateloupia doryphora* (García-Jiménez et al., 1998; Marián et al., 2000). However, further experiments with supplementation of individual and combinations of PA biosynthesis inhibitors such as DFMA, MGBG, AVG, and CHO (see full abbreviation in the legend of Figure 1) are needed to better illustrate which PA's biosynthetic pathway or which specific PA is involved in cell division to cell elongation during morphogenesis. It is also not clear, whether it is solely PAs that directly regulate growth and morphogenesis events or do they mediate some cross-talk with other phytohormones and proteins such as auxins and PIN proteins in regulating these developmental processes. Furthermore, recent reports showed positive effects of exogenous Spm in callus induction and micropropagule formation in *Kappaphycus alvarezii* (Muñoz et al., 2006; Neves et al., 2014) reinforcing the potential role of PAs in regulating morphogenesis and regeneration patterns in seaweeds. However, a precise mechanism for their mode of action is still unknown. It will be very interesting to explore, whether endo and/or- exogenous PAs regulate morphogenesis by promoting activation of proton pumps (H^+^-ATPase and H^+^-PPase) and nitric oxide (NO) generation. These compounds are generally associated with the regulation of cellular processes, such as enlargement of meristematic cells, cell elongation, embryogenesis as well as stress responses in plants (Dutra et al., 2013). Earlier speculations of passive PA uptake in seaweeds also need to be re-examined in the backdrop of recent report on the existence of L-type amino acid transporter (LAT) called *Resistant to Methylviologen 1* (*RMV1*) which is responsible for PA uptake in land plants. This finding emphasizes that for both prokaryotes and eukaryotes, PA transport is not just a passive mechanism, and selectivity could be achieved through specific recognition and translocation (Fujita and Shinozaki, 2014). Furthermore, over the past decade seaweed protoplast research has attained significant progress in establishing methods for generation of protoplast, their culture and subsequent plantlet regeneration from examples of red, brown and green seaweeds (Reddy et al., 2009). Protoplast cells are totipotent, meaning they have the capability of dedifferentiation, where they re-enter the cell cycle, go through repeated mitotic divisions and then proliferate or regenerate into various organs; this system could be useful for studying PA uptake and its involvement in seaweed developmental processes. Understanding about endogenous PAs regulation during cell cycle could provide insights into developmental patterns and mechanisms in seaweeds.

The importance of PAs in reproduction of seaweeds has been evident since the study of Guzmán-Urióstegui et al. (2002) who demonstrated a significantly higher level of PAs (especially Put) in immature cystocarps (reproductive tissue) of *Gracilaria cornea* as compared mature cystocarps. Further, enhanced carpospores release and transformation of infertile to fertile axes with the appearance of cystocarp when exogenously supplied with Spm (in contrast to Put and Spd) in different red seaweeds (Guzmán-Urióstegui et al., 2002, 2012; Sacramento et al., 2004, 2007) confirmed the potentials of PA in seaweed reproduction. These results indeed offer new opportunities to explore regulation of genes involved in PA biosynthetic pathways during cystocarp development and maturation events. However, these findings raise a critical question; why does only Spm (exogenous) apparently act as a sporulation-inducing agent; while all three PAs (Put, Spd, and Spm) were found to have accumulated during differential stages of cystocarp maturation? Recently, García-Jiménez and Robaina (2012) reported ethylene (ET) induced tetrasporogenesis mediated *via* up-regulation of Put biosynthesis in the red seaweed *Pterocladiella capillacea*. These findings suggest a synergistic action and possible cross-talk of ET and PAs in regulating reproductive processes and also could provide cross-talk between these metabolites and ET receptor responses at genetic level.

The potential function of Spm in seaweed development that has been reported so far, should actually be attributed to tSpm (a structural isomer of Spm) rather to Spm. It has been found that tSpm is present throughout the whole plant kingdom, while Spm is found only in angiosperms (Minguet et al., 2008) and thus the existence of Spm in seaweeds is questionable. Moreover, initial reports claimed that Spm synthase was encoded by two genes, *SPMS1* and *ACL5*; however, it has been demonstrated that *ACL5* (expressed in xylem vessel element) displays only tSpm synthase activity and tSpm plays a crucial role in regulating vascular development in *Arabidopsis* (Knott et al., 2007; Muniz et al., 2008). Therefore, the existence of Spm in seaweeds has been wrongly interpreted in earlier reports and should be replaced with tSpm. So far classical approaches based on HPLC and TLC used for Spm quantification have proven to be ineffective at distinguishing between Spm and tSpm. However, a recently developed method based on ion-pair extraction and gas chromatography–mass spectrometry (Rambla et al., 2010) has been most promising in clarifying the ambiguity with the existence and separation of tSpm from Spm and thus can also be used in seaweeds. Therefore, the functionality of Spm as an inducing agent for sporogenesis and carposporogenesis should be reinvestigated with exogenous application of tSpm, instead of Spm. It is possible that it is neither the Spm nor tSpm, rather a metabolic product of their oxidation that is responsible for maturation of reproductive structures, thus demanding further investigation. Apparently, the role of tSpm in land plants has been shown to influence stem elongation, vascular development, and cell wall patterning. However, as seaweeds lack a vascular system, it would be worth of exploring if gene encoding thermospermine synthase (*tSPMS*) expression is localized to a particular cell- or tissue-specific region such as sporangial and carposporangial cells in order to confirm its role in reproductive structure maturation and development. Knott et al. (2007) characterized *tSPMS* gene from the marine diatom *Thalassiosira pseudonana* and found it to be homologous to *Arabidopsis ACL5*. The regulation of *tSPMS* at transcriptional and metabolite levels (if induced by auxin or abscisic acid), and the influence of tSpm whether mediated *via* its oxidation or not during seaweed reproduction and development are both worthy of further examination. This may provide new insights into the role of PAs in metabolic pathways in response to developmental signals. Further, PA conjugates such as HCAAs (hydroxycinnamic acid amides-most abundant phenolic compounds in reproductive organs of plants) and their potential role in seaweed development is largely unknown.

## Molecular tools in hand for newer insights of PA's in reproduction and development of seaweeds

It is apparent from the previous studies that PAs are involved in seaweeds in some reproductive stages such as carposporogenesis, sporulation, and tetrasporogenesis. However, due to lack of extensive genomic information in seaweeds and the absence of authoritative databases on molecular gene expression studies, it is hard to establish functions of PAs as phytohormone. No molecular information is available on the number of genes encoding each of the specific enzymes involved in PA synthesis. The recent genome sequencing of *Ectocarpus siliculosus* (brown seaweed), *Chondrus crispus* and *Porphyra umbilicalis* (red seaweed), provided a new suite of opportunities to investigate phytohormone signaling, and diverse cellular gene expressions under different conditions in these model organisms (Kumar et al., 2014 and references therein). The functional genomics tools that are developed now for such studies in these seaweeds include RNA-seq data, EST-based expression microarrays, whole-genome tiling arrays, sRNAs sequences, a genetic map, bioinformatics, and proteomic tools (Kumar et al., 2014). Furthermore, a Japanese group (Mikami, 2013, 2014) has made excellent progress in establishing genetic transformation systems in the red algae *Porphyra* and *Bangiophycae* sp., which can serve as an excellent tool to transfer the gene expression constructs encoding proteins/enzymes involved in biosynthesis of different PAs. Development of mutants either by transposon, T-DNA (insertional mutagenesis) or by using sense and antisense transgenic approaches may facilitate the identification of knockouts with genes of interest and/ or gene regulations using PCR-based techniques that need to be develop in the near future to manipulate PA metabolism. PAs biosynthesis involves relatively few enzymes, therefore genes coding for respective enzymes should be characterized and cloned in model seaweeds to gain better insights into their role in reproduction and morphogenesis. Recently, cloning of ornithine decarboxylase gene from the red seaweed *Grateloupia imbricata* (*GiODC*) and its expression studies throughout the reproductive process provide evidence for PA's involvement in seaweed reproduction (García-Jiménez et al., 2009). It would be worthwhile to clone and characterize the gene *tSPMS* and elucidate its expression throughout the developmental stages to establish tSpm as an inducing agent for carposporogenesis and sporulation. Exploration of *SMADC4* (S-adenosyl methionine decarboxylase-if similar sequence exist in seaweeds) regulation during seaweed reproduction and development is also interesting as this gene has been suggested to be involve in tSpm synthesis. Moreover, Arabidopsis mutant *smadc4* exhibits vascular tissue alterations that partially resemble the *acl5* phenotype. Therefore, cloning and characterization of most of the genes involved in PA pathway and ET receptors in seaweeds may serve as an excellent model to test various hypotheses related to developmental processes in seaweeds derived from pathway manipulations. These breakthrough investigations are also equally important in establishing transgenic somatic embrogenic lines and studying PAs involvement in embryo development.

## Conclusions

The investigations conducted so far on PAs clearly show their potentials in regulating morphogenesis and developmental process in seaweeds. The opportunities that became available following whole genome sequences of *E*. *siliculosus*, *C*. *crispus*, and *P*. *umbilicalis* together with protoplast and somatic emryogenesis protocols, will enable the use of reverse genetics, transgenic, proteomic, and functional genomic approaches to generate critical new knowledge in understanding the role of PAs in seaweed developmental processes. Researchers should consider projects aimed at cloning and transforming genes encoding PA biosynthesis in seaweeds. The manipulation of endogenous PA levels in transgenic algal plants using tissue-specific or inducible promoters will greatly help in exploring the PA's functionality associated with various developmental processes in seaweeds.

### Conflict of interest statement

The authors declare that the research was conducted in the absence of any commercial or financial relationships that could be construed as a potential conflict of interest.
